# Annotations

**Published:** 1887-06-18

**Authors:** 


### Annotations.

It is a dreadful thing to be born a pauper: better be born an elephant, or a hedgehog, or a weasel. Civilisation shows certain splendours which dazzle the imagination, but it develops also some hideous excrescences which fill the thoughtful man with indignation. Who would be born a pauper if he could help it? What responsibility can little boys and girls—mere helpless infants—have in the matter? And yet how barbarously these poor little things are sometimes used by human fiends! The Holborn Guardians have been boarding out their pauper children—a very excellent plan. Many of the little ones have won their way into the hearts of their foster-parents, and have found homes and happiness with them. But some of the heartless creatures who have taken in the children have treated them with gross neglect and cruelty, in spite of their tender years and helpless condition. It is true there are no tales of unequalled horror to be related; and it is said that some of the stories really told are exaggerated. Granted; but what sort of men and women can they be who will treat any child other than kindly and tenderly? And the pauper child demands, at least from all instructed Christian hearts, especial sympathy and pity. He has come into a hard and selfish world; he must carry about with him always the brand of his pauper birth. Surely in the years of his simple and innocent infancy he might be allowed that measure of joyous freedom from trouble and care which the lambs and the young birds enjoy. Oh, for a Dante to make the world believe that a just retribution awaits all those who, either directly as foster-parents, or indirectly as guardians, "Offend these little ones" who are made in the image of God!

Who cares for the Pauper Children?

Middlesex, it appears, requires a new Lunatic Asylum large enough to provide for 2,000 additional patients. If there really be a necessity for such an additional asylum it must of course be built. Built it is evidently going to be, and at no inconsiderable cost. The architect's estimate for the building alone is no less a sum than £290,000; if to this be added the value of the site, furnishing, medical and surgical appliances and all kinds of general equipments needed at such an enormous institution, we shall probably not be far wrong in giving the total outlay at little less that £400,000. In addition to the cost of the buildings, the annual expenditure for the maintenance of such an establishment cannot but be enormous, and the burden upon the ratepayers will be correspondingly great. This, however, is the least important part of the question. What is the meaning of the terrible increase of lunacy among the pauper population? Is it possible to discover the cause, and if it can be discovered, can it be removed? We are always boasting of our scientific knowledge and our practical skill. Are there among us any men who can take such a problem as this in hand and solve it, and deliver the nation from the appalling nightmare of the huge and growing army of lunatics which is ever upon us? We are weary of talk and vain boasting, and would fain see a person of practical capacity who could deal with these tremendous social problems.

A New Lunatic Asylum for Middlesex.

Last Saturday there were found two thousand women ready and willing to bear the fatigues of a long day under a hot midsummer sun for the sake of the poor and the hospitals. How many more good lady Samaritans assisted and relieved the responsible two thousand we do not know. We sometimes speak rather hardly of women in these pages, especially of the frivolous and the extravagant; and we do this because we are so painfully conscious of the overwhelming amount of suffering and misery which might be relieved if some of the money now wasted on the most absurd trivialities could be put to charitable uses. But on this occasion we have nothing but praise and gratitude to bestow. Whether by right or no right, we have made the cause of hospitals our own, and whoever opposes or rebukes, we are minded to adhere to the lines on which we have set out. The hospitals for the poor, and the rich for the hospitals—that is our motto. Everyone, therefore, who helps them is to that extent our friend, and shall have at least our sincere and grateful thanks. The two thousand did nobly and well. We should have been pleased to record that their success had been ten times as great as it actually proved. The smallness of the amount, however, did not gauge the value of the work. Those valiant ladies brought home to the senses of busy Londoners the claims of the hospitals in a clear and unmistakable way. And if the actual amount collected was no more than five or six thousand pounds, the advertisement given to hospitals by the movement may yet bring them sixty, or even six hundred thousand. The education of the public is carried on by all manner of means, and we know of no copy in the social copy-book more pleasant to look upon and to imitate than that of thousands of kind-hearted young ladies spending their time and strength for the well-being of the sick poor. A question occurs to us as we think of the subject, viz., where were the young men? Could not two or three thousand club loungers have been enlisted for the service, and instead of having stalls at certain fixed points, have borrowed for the day a goodly number of movable stalls in the shape of costers' barrows? These they might have trundled into all sorts of little side-streets, and if they had made themselves exceedingly agreeable, who knows but what an additional thousand or two might have been added to the Fund? We offer this as a suggestion to all members of West End clubs who consider themselves still young—that is to say, who are on the right side of forty-five. We hope our suggestion will bear good fruit next year. Failing this, might not some of the more active spirits see that a list is suspended in each public room, and the names of a goodly number of members obtained, for liberal subscriptions to be sent through the Mansion House Fund?

Good Women.

